# Osteosarcopenia in Chronic Kidney Disease: An Overlooked Syndrome?

**DOI:** 10.1002/jcsm.13787

**Published:** 2025-04-07

**Authors:** Lara Caldiroli, Paolo Molinari, Claudia D'Alessandro, Adamasco Cupisti, Carlo Alfieri, Giuseppe Castellano, Simone Vettoretti

**Affiliations:** ^1^ Unit of Nephrology, Dialysis and Kidney Transplantation Fondazione IRCCS Ca’ Granda Ospedale Maggiore Policlinico di Milano Milan Italy; ^2^ Department of Clinical and Experimental Medicine University of Pisa Pisa Italy; ^3^ Department of Clinical Sciences and Community Health Università degli Studi di Milano Milan Italy

**Keywords:** chronic kidney disease (CKD), mineral bone disorder (MBD), osteoporosis, osteosarcopenia, sarcopenia

## Abstract

**Background:**

Healthy ageing relies on maintaining physiological systems, particularly the musculoskeletal system (MKS). After 50, declines in bone density, muscle mass and strength increase the risk of osteoporosis and sarcopenia, leading to frailty, fractures and higher healthcare costs. Osteosarcopenia, combining osteoporosis and sarcopenia, is rising because of the ageing population. Chronic kidney disease (CKD) exacerbates this condition through disruptions in mineral metabolism, hormonal imbalances and inflammation, further compromising musculoskeletal health.

**Aims:**

This review examines the pathophysiology of osteosarcopenia associated with CKD, focusing on the role of mineral and hormonal disturbances, chronic inflammation and endocrine dysfunction. It aims to increase clinical awareness and highlight the need for early diagnosis and intervention to mitigate the burden of osteosarcopenia on the quality of life and healthcare systems in ageing CKD populations.

**Methods:**

A narrative review of the current literature was conducted, summarising evidence on the mechanisms underlying osteosarcopenia in CKD, including mineral metabolism alterations, inflammatory processes and hormonal imbalances.

**Results:**

Osteosarcopenia is a recognised consequence of CKD, contributing to increased morbidity and mortality. The pathophysiology of osteosarcopenia in CKD is multifactorial, involving disruptions in mineral metabolism, inflammation, endocrine dysfunction and physical inactivity. CKD–mineral and bone disorder (CKD‐MBD) leads to alterations in calcium, phosphate, parathyroid hormone (PTH), fibroblast growth factor 23 (FGF‐23) and vitamin D metabolism, resulting in impaired bone mineralisation and increased fracture risk. Simultaneously, CKD accelerates muscle wasting through systemic inflammation, anabolic resistance and metabolic derangements, increasing the risk of sarcopenia. Sarcopenic obesity, inflammaging and hormonal dysregulation further exacerbate bone muscle deterioration. Emerging evidence suggests that osteosarcopenia in CKD is a consequence of interconnected pathophysiological pathways rather than isolated conditions. Diagnosis remains challenging because of overlapping clinical features, necessitating integrated assessment tools. Targeted therapeutic strategies, including mineral metabolism correction, resistance exercise and anabolic interventions, are essential to mitigate osteosarcopenia's progression and improve patient outcomes in CKD.

**Conclusions:**

Osteosarcopenia is a growing concern in ageing CKD populations. Early diagnostic strategies and targeted interventions are essential to mitigate the impact of osteosarcopenia on patient outcomes and reduce associated healthcare costs. Increased clinical awareness and research into effective therapies are crucial for improving the quality of life for individuals affected by CKD and osteosarcopenia.

## 1 Introduction

### 1.1 Osteosarcopenia: An Unrecognised Burden

The musculoskeletal (MSK) system plays a central role in enabling human ambulation and acts as a metabolic reservoir for calcium in bones and glucose and amino acids in muscles. However, in the sixth decade of life, a gradual decline in bone mineral density (BMD) (~1%–1.5% per year), muscle mass (~1% per year) and strength (~2.5%–3% per year) occur [1, 2], putting people at risk of osteoporosis and sarcopenia—two conditions classified by the International Classification of Diseases.

Osteopenia and osteoporosis are defined by the World Health Organisation as *T*‐scores of −1 and −2.5 standard deviations below the peak bone mass of a young healthy population, respectively [1, 3]. Osteoporosis reduces the micro‐architecture of the bone and impairs bone strength [3, 4], whereas sarcopenia is characterised by low muscle strength, low muscle mass and quality, and its severity is defined by the degree/or low functional capacity [1, 4]. Both conditions share common risk factors [1, 3]. They are strongly associated with frailty, falls, fractures, hospitalisation and mortality, leading to increased healthcare expenditure [1, 5].

The term osteosarcopenia, coined by Duque and colleagues, describes a subgroup of older people affected by both osteoporosis and sarcopenia [1, 6]. The combination of low bone density (osteopenia/osteoporosis) and low muscle mass, strength and/or functional capacity (sarcopenia) defines this unique syndrome [3].

With an ageing population expected to increase from ~841 million in 2013 to ~2 billion by 2050 (a proportional increase of 9%), the prevalence of osteosarcopenia is expected to increase, leading to increased incidence of falls, fractures and hospitalisations [1, 7].

Muscle and bone loss often occur together in older people, and many studies have shown a strong relationship between the components (osteoporosis and sarcopenia) of osteosarcopenia [8]. In a cohort of 590 postmenopausal Finnish women, those with sarcopenia had a 12.9 times higher risk [95% confidence interval (CI) 3.1–53.5] of having osteoporosis than those without sarcopenia [9]. In the Sarcophage cohort of 232 older people, those with sarcopenia had a five times higher risk of developing osteoporosis [95% CI 1.16–19.41]. Subsequent cross‐sectional and longitudinal studies have provided further support for the bidirectional relationship between osteoporosis and sarcopenia, leading to the development of osteosarcopenia [10, 11].

### 1.2 The Significance of Osteosarcopenia in CKD

Advanced age is burdened not only by a significant impairment in functional capacity, as described above, but also by the frequent onset of multiple comorbidities. In this context, CKD is a condition that predominantly affects elderly individuals and is frequently associated not only with a significant burden of comorbidities but also with a substantial impairment of the patient's functional status.

CKD is a complex disease characterised by progressive renal dysfunction. The impact of CKD extends beyond renal function to multiple organ systems, including the MSK system.

Among the various systems affected by CKD, the metabolic trophism, osteomuscular health and functional reserve of the patient are among the most important. However, there are currently no clear guidelines and/or expert consensus to guide clinical decision‐making.

This review aims to provide clinicians with an overview of the pathophysiology and diagnosis of osteosarcopenia in chronic kidney disease (CKD) in order to raise awareness of this under‐recognised MSK syndrome.

## 2 Pathophysiology of Osteosarcopenia in CKD

Osteosarcopenia is a recognised consequence of CKD and contributes to increased morbidity and mortality. The pathophysiology of osteosarcopenia in CKD involves a complex interplay between hormonal, inflammatory and metabolic factors.

### 2.1 CKD–Mineral Bone Disorder (CKD‐MBD)

**FIGURE 1:**
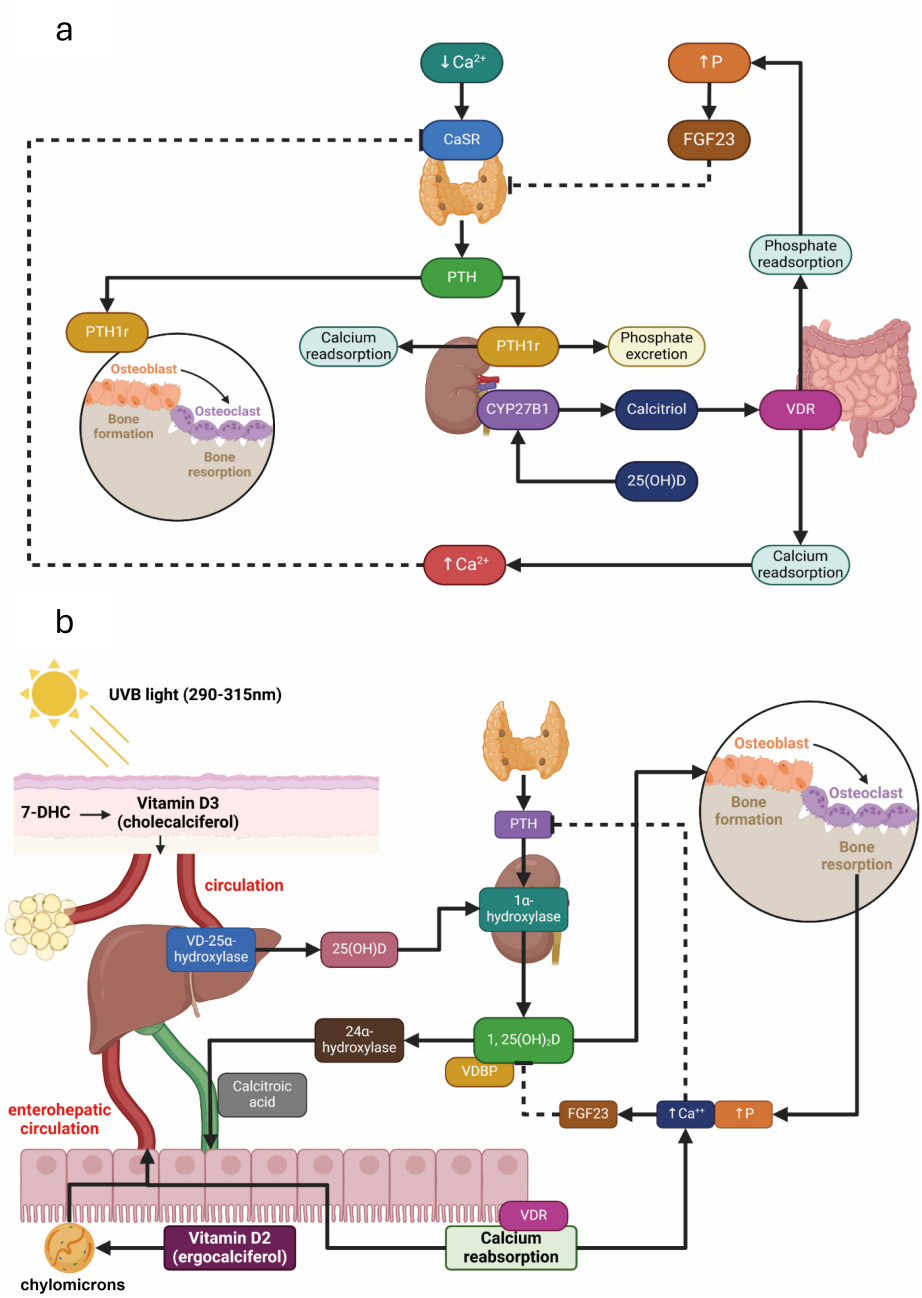
Schematic representations of PTH, vitamin D and FGF‐23 metabolism. *Note:* Schematic representations of PTH, vitamin D and FGF‐23 metabolism, illustrating their integrated roles in maintaining calcium‐phosphate homeostasis. The diagram details the mechanisms of PTH secretion in response to changes in serum calcium, its effects on bone resorption and renal phosphate excretion. It also highlights vitamin D metabolism, including its synthesis from precursors through sunlight and dietary sources, conversion to 25OH‐D in the liver and activation to 1,25OH‐D in the kidneys, emphasising its role in calcium absorption in the gut and feedback regulation of PTH. Additionally, the figure depicts FGF‐23 secretion by osteocytes in response to hyperphosphatemia, its interactions with the Klotho coreceptor and its suppression of calcitriol synthesis and renal phosphate reabsorption. These pathways collectively contribute to the dynamic regulation of mineral metabolism, with dysregulation contributing to the pathophysiology of CKD‐MBD. 1,25(OH)_2_D, 1,25(OH) vitamin D; 25(OH)D, 25(OH) vitamin D; 7‐DHC, 7 dehydrocholesterol; Ca, calcium; CaSR, calcium sensing receptor; FGF‐23, fibroblast growth factor 23; P, phosphate; PTH, parathormone; PTH1r, parathormone 1 receptor; VDR, vitamin D receptor.

CKD is a complex condition involving biochemical changes in the metabolism of calcium, phosphorus, parathormone (PTH), vitamin D and fibroblast growth factor 23 (FGF‐23). The main aspects of mineral metabolism are depicted in Figure 1 [12, 13].

#### 2.1.1 PTH Axis in CKD

The regulation of extracellular calcium levels is essential for cellular functions, with calcitriol controlling calcium absorption in the gut. Calcium directly affects parathyroid cells via the calcium‐sensing receptor (CaSR), which detects changes in extracellular calcium levels. A decrease triggers parathyroid hormone (PTH) release, whereas an increase suppresses it. Bone changes in CKD are linked to early disruptions in phosphorus, calcium, PTH and calcitriol metabolism as renal function deteriorates [14, 15]. In the proximal tubule, PTH inhibits phosphate reabsorption by reducing the abundance of sodium phosphate cotransporters in the apical membrane. PTH likely decreases calcium reabsorption from the proximal tubule, by reducing the reabsorption of sodium, an event necessary for the paracellular movement of calcium across this segment. In the thick ascending limb (TAL), PTH increases calcium permeability and may increase the electrical driving force thereby increasing calcium reabsorption in the TAL. These mechanisms maintain calcium and phosphate balance until GFR declines significantly, after which hyperphosphatemia occurs [14, 16].

#### 2.1.2 Vitamin D in CKD

Vitamin D metabolism relies on sunlight and dietary intake. Sunlight generates cholecalciferol, which is converted to 25OH‐D in the liver and then hydroxylated in the kidney to form active vitamin D (1‐25OH‐D), critical for metabolic functions [17]. CKD patients, even in early stages, show vitamin D deficiency due to interstitial fibrosis and reduced kidney synthesis of 1‐25OH‐D, inhibited by elevated FGF‐23 and phosphate levels [13, 14]. Calcitriol deficiency impairs calcium absorption, decreases systemic VDR levels and reduces CaSR expression in parathyroid cells, further stimulating PTH secretion. Elevated serum phosphorus also exacerbates PTH secretion via CaSR interaction [14, 18]. This creates a feedback loop contributing to secondary hyperparathyroidism. PTH enhances bone turnover through RANK‐L and osteoprotegerin signalling, leading to weakened, fragile bones and increased fracture risks, along with vascular calcifications [14, 15].

Vitamin D deficiency also impacts MSK health. Experimental vitamin D deficiency or VDR knockout models reveal muscle atrophy, reduced mass and impaired function [19, 20]. Overexpression of VDR induces muscle hypertrophy, with VDR levels increasing following resistance training [21]. VDR deletion disrupts metabolic pathways and neuromuscular control, exacerbating risks for osteosarcopenia, falls and fractures [22]. These findings highlight the intricate links between vitamin D, bone health and CKD progression.

#### 2.1.3 FGF‐23 in CKD

FGF‐23's role in bone metabolism remains complex. It suppresses calcitriol synthesis and regulates phosphate excretion, influencing bone abnormalities in CKD [3, 14, 23]. FGF‐23 levels increase before phosphate levels rise [12, 13, 16] and correlate with bone mineralisation changes. Studies suggest that FGF‐23 levels rise early in CKD, even before PTH [15, 16, 24]. It stimulates osteoblast‐like cell proliferation while inhibiting mineralisation via its coreceptor Klotho [12, 25]. In dialysis patients, high bone turnover associates with elevated serum FGF‐23, yet high FGF‐23 correlates with normal mineralisation. Regression analysis identifies FGF‐23 as an independent predictor of mineralisation lag time [12, 26]. Paediatric CKD patients with high bone turnover and renal osteodystrophy also exhibit improved mineralisation linked to high FGF‐23, though bone formation rates remain unaffected [12].

Elevated FGF‐23 levels align with high bone turnover, whereas low levels associate with reduced turnover [12]. These findings position FGF‐23 as a predictor of bone metabolism and mineralisation changes in CKD patients undergoing dialysis.

PTH and FGF‐23 also interact directly within the bone. PTH increases FGF‐23 mRNA levels and inhibits sclerostin mRNA in osteoblast‐like cells, implicating the Wnt pathway in their actions.

#### 2.1.4 Other Bone Metabolism Biomarkers in CKD

Protein expression studies in CKD patients reveal that as CKD progresses, serum alkaline phosphatase, FGF‐23, PTH and osteoprotegerin increase while calcium decreases, leading to heightened bone resorption and reduced mineralisation [12, 27]. Sclerostin, a marker of mature osteocytes, is expressed more in early‐stage CKD, whereas FGF‐23 expression predominates in early osteocytes. Sclerostin levels rise early in CKD, even before FGF‐23 or PTH elevation, as shown in the jck mouse model of progressive kidney disease. Sclerostin negatively correlates with bone formation, osteoblast function and PTH levels in stage 5D CKD patients [12, 15]. Mice and renal osteodystrophy patients exhibit an inverse correlation between sclerostin and PTH levels, with sclerostin predicting low bone turnover and contributing to adynamic bone disease and fractures [28, 29].

Moreover, increased PTH stimulates osteoblasts and osteoclasts via the receptor activator of nuclear factor‐kappaB (RANK‐L) and osteoprotegerin signalling pathways, leading to increased bone turnover, resulting in a less strong and more fragile bone structure, which contributes to increased fracture risk and changes in vascular metabolism leading to vascular and valvular calcification [14, 15].

### 2.2 CKD Affects Both Bone and Muscle Health

Patients with CKD and low bone turnover are often older individuals with a high prevalence of comorbidities, including malnutrition, inflammation and hypoalbuminemia. These factors contribute to the development and progression of low bone turnover, a condition marked by a lack of proper bone remodelling activity. Several underlying mechanisms and conditions predispose CKD patients to low bone turnover, including insufficient PTH signalling, diminished WNT/β‐catenin signalling pathways, malnutrition, systemic inflammation, diabetes mellitus and the presence of uremic toxins (Figure 3) [29, 30]. These disruptions collectively impair bone formation and resorption, leading to skeletal fragility and increased susceptibility to fractures.

Osteoporosis, falls and fractures are particularly common in CKD patients because of abnormalities in calcium, phosphate, PTH and vitamin D metabolism. These disturbances, coupled with cardiovascular calcifications and structural bone abnormalities, contribute to a heightened risk of fractures and other complications [31, 32]. CKD stages 3a‐5D are characterised by reduced BMD and mechanical strength, which significantly increases the likelihood of fractures. For individuals with end‐stage kidney disease (ESKD), the fracture risk is two to three times higher than in the general population [6, 33, 34]. Moreover, fractures in CKD patients tend to occur at a younger age compared with the general population, reflecting the accelerated bone deterioration associated with the disease [31, 35]. The consequences of these fractures are severe, as CKD patients experience significantly higher morbidity and mortality rates. For example, the mortality risk for dialysis patients is approximately 3.7 times greater than that of the general population [31, 36].

In addition to bone health issues, CKD also significantly impacts skeletal muscle mass. Patients with CKD frequently experience muscle wasting because of increased protein catabolism and reduced protein synthesis, processes exacerbated by uraemia and systemic inflammation [33, 37]. Although muscle mass naturally declines with age, individuals undergoing dialysis face an accelerated rate of muscle loss, surpassing what would typically be expected from ageing alone [38, 39]. This reduction in muscle mass is compounded by frailty and diminished physical activity, further increasing the risk of falls and fractures in this vulnerable population [39, 40, 41]. The dual impact of impaired bone and muscle health highlights the importance of addressing both conditions in CKD patients.

Osteosarcopenia, a syndrome defined by the co‐occurrence of osteoporosis and sarcopenia, is increasingly recognised as a critical factor in the heightened fracture risk among CKD patients. However, the concept of osteosarcopenia remains relatively new, and most studies investigating its role in falls and fractures have focused on the general population.

### 2.3 Sarcopenic Obesity

The prevalence of sarcopenic obesity in CKD is reported to be 2%–23% [42].

Baumgartner first proposed the term ‘sarcopenic obesity’ in 2000, defining it as a phenotype in which sarcopenia and excess fat, that is, obesity, coexist [43, 44]. Sarcopenic obesity is an emerging geriatric syndrome that leads to many adverse clinical complications such as frailty, falls, disability, immobility, fractures, cardiometabolic and respiratory diseases, cancer and increased mortality [45, 46, 47]. Sarcopenia and obesity are independently associated with the risk of falling, and the risk of fracture is increased in older men with sarcopenic obesity compared with men who are not sarcopenic obese [19, 48]. In addition, obese people have relative BMD deficiency and low bone turnover associated with insulin resistance [19, 49], creating the trinity of osteosarcopenic obesity [19, 50].

Adipogenesis was found to be an expression of biological ageing via the mesenchymal stem cell (MSC) differentiation pathway [33, 51]. Important stages in the development of osteoporosis may be related with the presence of medullary adiposity and/or intramuscular adipose tissue. The presence of adipocytes can have an impact on the microenvironment by altering myogenesis and osteogenesis producing local levels of adipokines, free fatty acids and lipids. This can lead to local lipotoxicity, with a decrease in bone formation and an increase in bone resorption [33, 52].

As a result, the infiltration of fat into muscle fibres has been correlated with cellular dysfunction [33, 53]. In muscle, fat can accumulate between muscle bundles in the form of adipocyte deposits (intramuscular fat) and/or within muscle fibres in the form of fat infiltrates (intramyocellular fat). These infiltrations appear to involve insulin resistance, inflammation and skeletal muscle dysfunction [33, 54, 55].

CKD contributes to the development of sarcopenic obesity. This is driven by several interrelated mechanisms affecting muscle and adipose tissue metabolism. CKD‐associated insulin resistance impairs glucose uptake by muscle cells, leading to muscle atrophy while promoting fat storage, particularly in visceral adipose tissue [56, 57]. CKD is also characterised by a state of low‐grade chronic inflammation, driven by elevated levels of pro‐inflammatory cytokines such as IL‐6 and TNF‐α. These cytokines promote muscle breakdown (catabolism) while simultaneously stimulating fat accumulation by inducing insulin resistance and promoting adipogenesis [58].

CKD disrupts several hormonal axes, including reduced levels of anabolic hormones (growth hormone, testosterone and IGF‐1) and altered adipokines (leptin and adiponectin), and also contributes to muscle loss and fat accumulation. In addition, protein‐energy wasting (PEW), a common complication of CKD, exacerbates muscle wasting while fat mass is maintained or increased [57].

### 2.4 Inflammageing

**FIGURE 2:**
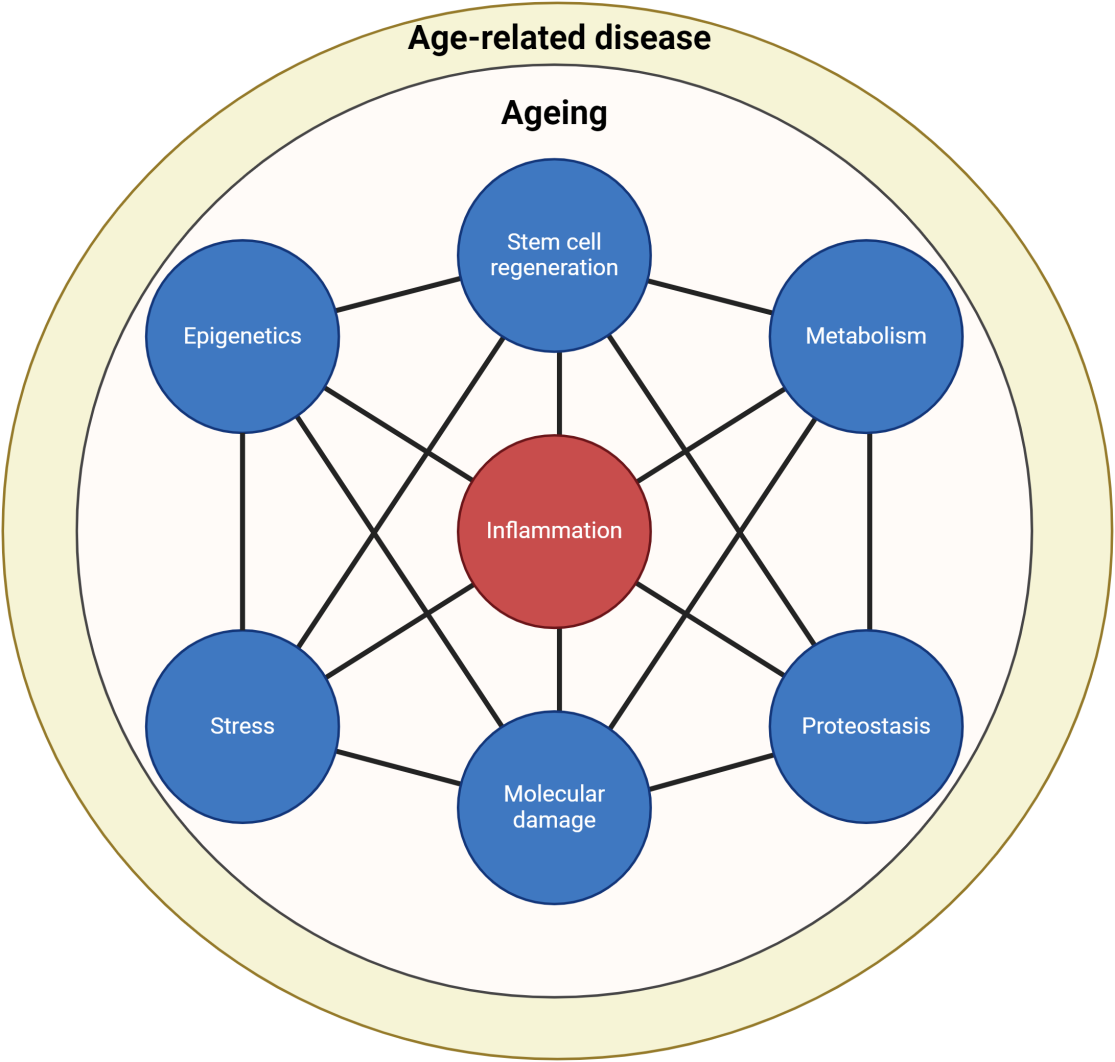
Cornerstones of inflammaging. *Note:* Inflammaging, a portmanteau of inflammation and ageing, describes the chronic, low‐grade inflammation prevalent in aged individuals. This figure illustrates the complex interplay of various cellular and molecular components contributing to inflammaging. Elevated levels of pro‐inflammatory cytokines, such as interleukin‐6 (IL‐6) and tumour necrosis factor‐alpha (TNF‐α), alongside activated immune cells like macrophages and T cells, signify the inflammatory milieu characteristic of inflammaging. This sustained inflammatory state is implicated in the pathogenesis of age‐related diseases, including cardiovascular disorders, neurodegenerative conditions and metabolic syndromes, ultimately influencing overall health span and longevity.

CKD is associated with PEW, a condition characterised by the loss of body weight including lean muscle mass. The aetiology of PEW in CKD is complex and multifactorial: reduced food intake because of anorexia, changes in taste, uremic gastritis and the high number of medicines prescribed may all contribute to reducing energy and protein intake [59, 60]. PEW is also associated with increased production of pro‐inflammatory cytokines, in particular IL‐6, IL‐1 and TNF, and the resulting inflammatory state leads to loss of bone and muscle mass. The term ‘inflammaging’ emerged in 2000 to describe a chronic, low‐grade inflammation that increases with age and is a significant risk factor for morbidity and mortality in elderly individuals (Figure 2) [61, 62]. This age‐related increase in background inflammation is thought to result from cumulative exposure to environmental and infectious antigens, leading to the production of reactive oxygen species (ROS). ROS stimulate further innate and adaptive immune cytokine release, shifting the immune balance towards chronic inflammation [61, 63]. Studies have linked chronically elevated inflammatory cytokines to the development of sarcopenia, possibly by activating the ubiquitin‐protease pathway [62, 64, 65] and by increasing pro‐inflammatory cytokines such as TNF‐a, IL‐1 and IL‐6, which promote bone resorption [62, 64, 66]. These cytokines promote osteoclastogenesis, the process by which osteoclasts (bone‐resorbing cells) are formed. The increased osteoclast activity leads to excessive bone resorption, weakening bone structure and increasing fracture risk [62, 64, 66]. The chronic inflammatory state disrupts the delicate balance between bone formation and resorption, leading to bone loss characteristic of osteoporosis and osteosarcopenia. Impaired chronic inflammation also interferes with bone regeneration processes. Inflammatory cytokines reduce the activity and survival of osteoblasts, limiting their capacity to repair and regenerate bone tissue. This impaired bone remodelling contributes to reduced bone density and increased susceptibility to fractures, particularly in elderly CKD patients. In addition, epidemiological studies have shown a positive association between both osteoporosis and sarcopenia and C‐reactive protein (CRP), which is a marker of active inflammation [67, 68, 69, 70]. One known mechanism of communication between the bone and muscles is through factors called myokines, which are released from muscle, and osteokines, which are released from bone, such as osteocalcin. One myokine, myostatin, has been extensively studied. In mice, it has been shown to play an important role in the impaired proliferative capacity of muscle and bone progenitor cells with age [71]. In addition, it has been shown that the Wnt‐b‐catenin pathway mediates the cross‐talk between bone and muscle by controlling both osteogenesis and muscle regeneration [72]. Understand the molecular pathways through which muscle and bone work together offers potentially exciting molecular targets for developing therapies to treat osteosarcopenia.

### 2.5 Endocrine Dysfunction

Anabolic hormone deficiency may be a contributory factor in the development of MSK disorders.

In particular, hormones associated with the development of sarcopenia and bone loss in CKD are testosterone, myostatin and growth hormone/insulin‐like growth factor‐1 (IGF‐1) [73]. The prevalence of hypogonadism in men with CKD was reported to be 46.4% [74]. When haemodialysis and nondialysis‐dependent CKD (NDD‐CKD) male patients were compared with healthy controls, testosterone levels and muscle mass were lower in dialysis‐dependent patients [74]. The release of myostatin in muscle cells has been linked to inflammation and oxidative stress. Decreased testosterone leads to increased myostatin expression in muscle cells and impaired IGF‐1 signalling [75]. This has been suggested to have a negative effect on muscle differentiation and growth by stimulating protein catabolism and reducing protein synthesis [75]. Resistance to the growth hormone/IGF‐1 axis leads to low muscle mass and is associated with sarcopenia in end‐stage renal disease (ESRD) because of reduced renal clearance of growth hormone, metabolic acidosis, inflammation and uraemia [73]. Conditional deletion of the androgen receptor in satellite cells or fast‐twitch muscle fibres further contributes in an osteosarcopenic phenotype [19, 76, 77].

Androgens are also known to determine periosteal bone expansion during puberty via the androgen receptor [19, 78]. Through the androgen receptor in osteoblasts and osteocytes, but not in osteoclasts, androgens also exert antiresorptive effects in bone [76, 79]. Indirectly, the androgen receptor in neuronal cells also prevents the thinning of the cortical bone and the loss of trabecular bone in the vertebral body in mice [80]. It is interesting to note that androgen deficiency increases the skeletal response to mechanical stress [79].

Oestrogen plays a crucial role in the maintenance of both bone and muscle health, and its deficiency has been implicated to a significant degree in the pathogenesis of osteosarcopenia, particularly in patients with CKD [81, 82]. Oestrogen is critical for bone homeostasis, primarily through its effects on bone remodelling. It regulates the balance between bone resorption and bone formation by acting on osteoclasts, osteoblasts and osteocytes. Oestrogen suppresses osteoclastogenesis by downregulating receptor activator of nuclear factor κB ligand (RANKL), a key promoter of osteoclast differentiation, while increasing osteoprotegerin (OPG), a decoy receptor for RANKL. This hormonal action helps to maintain bone mass by limiting excessive bone resorption [81, 83]. In the absence of oestrogen, such as in postmenopausal women or CKD patients with endocrine dysfunction, increased bone resorption occurs, leading to reduced BMD and increased risk of fracture [82]. This problem is compounded by the development of CKD‐MBD, which is associated with dysregulation of calcium, phosphate and PTH levels [82]. Oestrogen deficiency exacerbates this condition and accelerates bone loss. In addition, CKD patients are prone to secondary hyperparathyroidism due to impaired renal function, leading to elevated PTH levels, which further increase bone resorption. Studies have shown that oestrogen replacement therapy (ERT) can counteract this process by reducing PTH levels and markers of bone turnover, ultimately improving BMD and reducing the risk of fracture in postmenopausal women with CKD [82].

In addition, oestrogen also affects muscle mass, which is critical in the context of osteosarcopenia. Oestrogen deficiency contributes to sarcopenia through multiple mechanisms, including impaired muscle protein synthesis, increased inflammation and mitochondrial dysfunction. Oestrogen has been shown to increase muscle protein synthesis by modulating the expression of insulin‐like growth factor 1 (IGF‐1), a key regulator of muscle anabolism. In CKD patients, where PEW and inflammation are prevalent, the protective effects of oestrogen on muscle mass are further reduced, exacerbating sarcopenia [81, 82]. Oestrogen also has anti‐inflammatory effects on pro‐inflammatory cytokines such as tumour necrosis factor‐alpha (TNF‐alpha) and interleukin‐6 (IL‐6), both of which are elevated in CKD and contribute to muscle wasting and bone loss. In postmenopausal women with CKD, ERT has been associated with improvements in both bone and muscle parameters [81, 82]. In addition to increasing BMD, oestrogen therapy improves muscle strength and reduces the risk of falls and fractures, which are key factors in preventing osteosarcopenia‐related morbidity and mortality. However, the use of ERT in CKD must be carefully considered because of potential risks such as thromboembolic events and cardiovascular complications, especially in people with advanced CKD [81, 82].

### 2.6 Osteosarcopenia and Physical Activity in CKD Patients

The relationship between physical activity and osteosarcopenia in CKD is complex and multifaceted, involving interactions between muscle and bone metabolism, systemic inflammation and hormonal dysregulation [84]. Physical activity, particularly resistance exercise, has been shown to play a critical role in mitigating the effects of osteosarcopenia by improving both bone and muscle health [84, 85]. One of the central mechanisms by which physical activity influences osteosarcopenia in CKD is by promoting mechanical loading of bone and muscle. Mechanical stimuli are critical for maintaining bone mass and structure by stimulating osteoblast activity and suppressing osteoclast‐mediated bone resorption, thereby improving BMD. Physical activity has been associated with increased BMD, which is essential for reducing fracture risk in CKD patients who are already vulnerable to skeletal fragility due to CKD‐MBD [86].

In addition to bone health, physical activity is essential for maintaining and increasing muscle mass [87]. CKD patients often experience sarcopenia due to PEW, chronic inflammation and hormonal [87] imbalances, all of which contribute to muscle wasting. Resistance exercise has been shown to counteract muscle wasting by promoting muscle protein synthesis and improving muscle strength, which is particularly important in preventing falls and fractures in patients with CKD and osteosarcopenia [86, 87]. Exercise induces muscle growth by activating the mechanistic target of rapamycin (mTOR) pathway, which regulates muscle protein synthesis, and by inhibiting myostatin, a negative regulator of muscle growth [87].

Physical activity also has anti‐inflammatory effects that are beneficial in the context of osteosarcopenia and CKD. CKD patients have elevated levels of pro‐inflammatory cytokines such as IL‐6 and TNF‐α, which contribute to both muscle wasting and bone loss [88]. Regular physical activity has been shown to reduce inflammation by modulating cytokine production, which in turn helps maintain muscle mass and bone integrity. The anti‐inflammatory effects of exercise are particularly important in patients with CKD, where ‘inflammaging’ exacerbates the progression of osteosarcopenia [88].

The benefits of physical activity go beyond direct improvements in bone health. Improved physical function and mobility resulting from exercise interventions can improve the overall quality of life of people with CKD, reduce the risk of falls and increase independence [88]. Given the progressive nature of osteosarcopenia in CKD, early and sustained physical activity interventions are essential to delay the onset of severe functional impairment [88].

In conclusion, physical activity has a pivotal role in mitigating the deleterious effects of osteosarcopenia in CKD through the promotion of bone formation, the increase of muscle mass and the reduction of inflammation. Given the high prevalence of sarcopenia and osteoporosis in patients with CKD, the incorporation of structured exercise programmes, particularly resistance training, into the management of CKD may have a significant therapeutic benefit.

## 3 Diagnosis

### 3.1 Diagnosis of Osteoporosis

The gold standard to assess bone mass is dual X‐ray absorptiometry (DXA). The diagnosis of osteoporosis is made when any of the following criteria is met [89, 90]:

- History of fractures;
- *T*‐score ≤ −2.5 at the lumbar spine, femoral neck, total hip or distal 1/3 radius on DXA exam;
- *T*‐score between −1.0 and −2.5 with elevated fracture risk as determined by the Fracture Risk Assessment Tool (FRAX) that combine BMD with clinical risk factors [91]

There is general consensus on the fact that DXA should be performed in postmenopausal women aged ≥ 65 years regardless the presence or not of risk factors, man aged 70 years or more and younger subjects aged 50–65 years in the presence of risk for fracture, previous fragility fractures, family history of fractures, steroid therapy (daily dose equal or higher than 5 mg), alcohol consumption, smoking habit, presence of diseases that expose to the risk of fractures [89, 92].

In addition, it is essential to screen all patients at risk for secondary osteoporosis. Among these, patients with CKD are at particularly high risk because of osteomalacia resulting from secondary hyperparathyroidism on one hand and vitamin D deficiency induced by renal insufficiency on the other [93].

However, in patients with CKD, the interpretation of DXA results is more complex because of kidney disease‐specific factors:

1. Altered bone mineralisation: CKD is associated with impaired bone mineralisation due to impaired phosphate balance, elevated FGF‐23 and secondary hyperparathyroidism. These factors can reduce bone quality, and altered BMD measurements may not accurately reflect bone strength or fracture risk. In particular, patients with CKD may have low turnover bone disease (adynamic bone disease), where BMD may appear normal or even elevated despite reduced bone strength [94, 95].
2. Vascular calcification: Vascular calcification is common in CKD patients and can interfere with DXA scans. Calcification of arteries and soft tissues can lead to falsely high BMD measurements and mask the true extent of osteoporotic bone loss [96].
3. Variability in bone turnover: CKD‐BD encompasses a range of conditions from high bone turnover (osteitis fibrosa) to low turnover (adynamic bone disease). These conditions may present normal or increased BMD but involve significant structural abnormalities of the bone. Advanced imaging techniques and biochemical markers of bone turnover, including PTH, alkaline phosphatase and markers of bone resorption (e.g., C‐telopeptide), are essential for accurate diagnosis and treatment decisions [97].

### 3.2 Diagnosis of Sarcopenia

According to the European Working Group on Sarcopenia in Older People 2 (EWGSOP2), sarcopenia can be defined according to three criteria: reduction of muscle strength, reduction of muscle mass and muscle quality and reduction of functional capacity [98].

The first criterion, low muscle strength, identifies probable sarcopenia. The second criterion, low muscle mass or quality, confirm the diagnosis. The simultaneous presence of the three criteria determines the severity of the sarcopenia condition.

Figure 4 shows the cut values for each criterion suggested by the EWGSOP2.

Muscle strength can be assessed using a handheld dynamometer, an inexpensive and non‐invasive tool suitable for daily clinical practice. Alternatively, the chair test (sit‐to‐stand test with five repetitions) can be used to evaluate lower limb strength. For muscle mass evaluation, dual‐energy X‐ray absorptiometry (DXA), computed tomography (CT) and magnetic resonance imaging (MRI) are the gold standards. However, their use is often limited to research settings. Bioimpedance offers a practical, non‐invasive and cost‐effective bedside alternative, quantifying muscle mass as appendicular skeletal mass (ASM) in absolute terms or normalised for height (ASM/m^2^).

Emerging in the nephrological‐nutritional field, muscle ultrasound (US) is a portable and non‐invasive technique that can be applied in routine clinical practice by trained healthcare professionals. Muscle US correlates well with gold‐standard methods like MRI and CT in detecting changes in muscle volume and structure [99, 100]. The anterior thigh, particularly the rectus femoris (RF) muscle, is a preferred site for US measurements, as it represents the largest muscle group crucial for patient mobility. Quadriceps thickness correlates with muscle strength and remains measurable even in severe muscle depletion [101].

**FIGURE 3:**
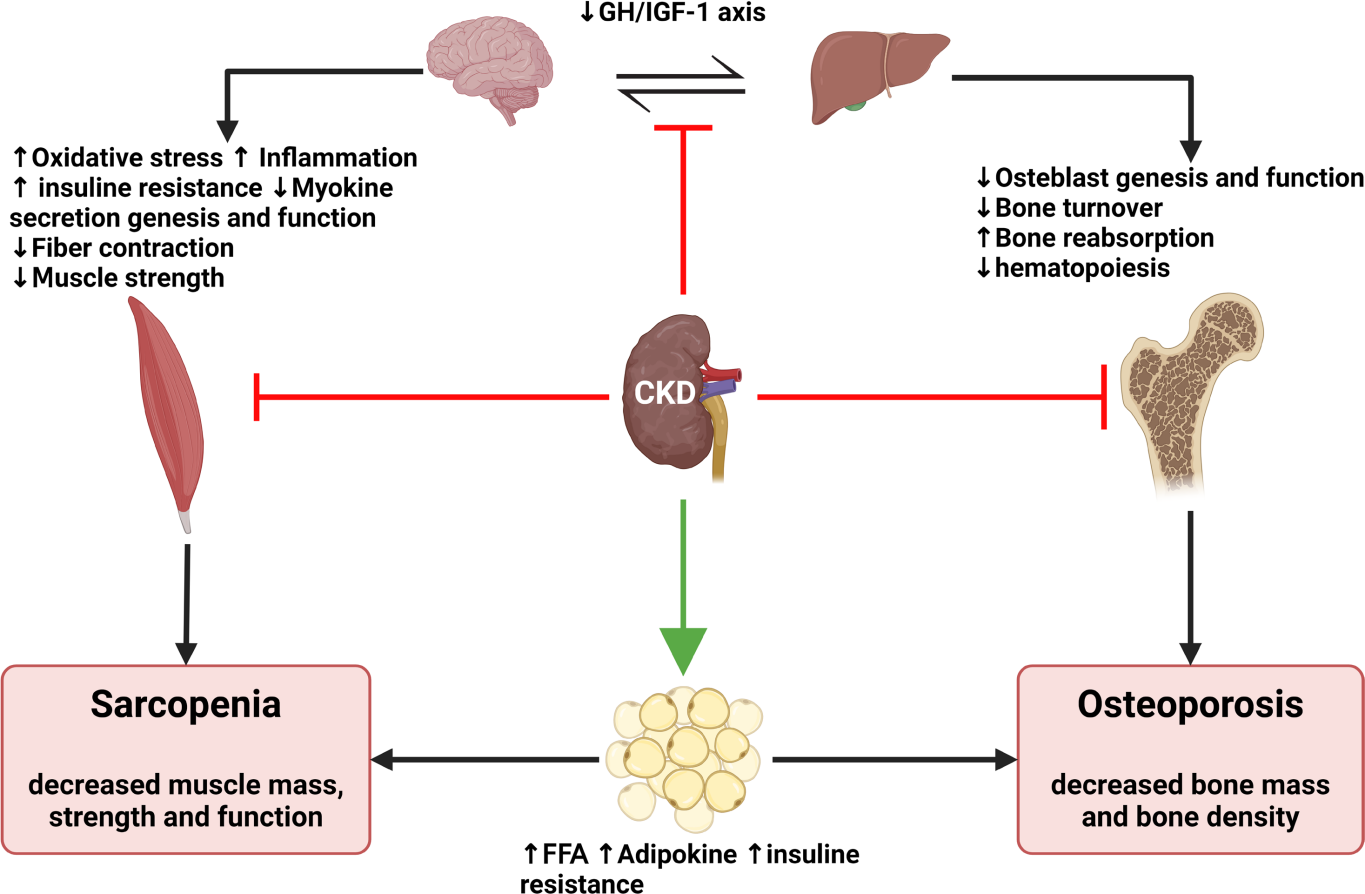
Main factors associated with osteosarcopenia development. *Note:* This figure depicts the multifactorial pathways leading to osteosarcopenia development in chronic kidney disease (CKD). Reduced renal function in CKD disrupts mineral and hormonal homeostasis, resulting in secondary hyperparathyroidism, elevated fibroblast growth factor 23 (FGF‐23) and decreased vitamin D synthesis. These disturbances contribute to bone loss, osteoporosis and increased fracture risk. Concurrently, CKD‐induced inflammation, metabolic acidosis and uremic toxins impair skeletal muscle integrity, promoting sarcopenia. The reciprocal relationship between bone and muscle further exacerbates the progression of osteosarcopenia, leading to frailty, disability and heightened mortality risk in CKD patients. GH, Growth hormone; IGF, Insuline growth factor; CKD, Chronic Kidney Disease; FFA, Free fatty acids.

US also provides insights into muscle quality, now included in the revised European consensus on sarcopenia diagnosis [98]. Echo intensity (EI), a key US parameter, reflects muscle texture and fat infiltration [102]. S. Wilkinson et al. demonstrated correlations between US‐derived muscle texture parameters and MRI findings in CKD patients, highlighting the potential of US in identifying muscle mass reduction and increased fat infiltration [103]. For further details, see Figure 3.

The diagnosis of sarcopenia in CKD is particularly challenging because of the complex interplay between muscle wasting and renal dysfunction. CKD‐specific factors like chronic inflammation, metabolic dysfunction and PEW contribute to muscle catabolism, complicating sarcopenia diagnosis [104]. Elevated inflammatory markers, such as CRP and IL‐6, exacerbate muscle loss and can make it difficult to distinguish sarcopenia from other conditions. In addition, PEW, which is the result of inadequate protein intake and metabolic imbalances, has overlapping diagnostic criteria with sarcopenia, which further complicates the clinical assessment [105].

Diagnostic tools that have traditionally been used for sarcopenia, such as BIA and DXA, may be unreliable in patients with CKD due to fluid imbalances, such as oedema, which can confound measurements of muscle mass. More advanced imaging techniques, including MRI and CT scans, may provide more accurate assessments in this population [106]. In addition, patients with CKD often experience a decline in physical function and frailty, which is associated with sarcopenia but may also be influenced by other factors such as anaemia and cardiovascular comorbidities, further complicating the diagnosis [107].

### 3.3 Diagnosis of Osteosarcopenia

In the literature, there is very limited data regarding a precise definition of osteosarcopenia, particularly in the context of CKD. Our goal in this section is to provide the clearest possible guidance that can be utilised by both clinicians and researchers to assess and define this condition in their patients. However, it should be noted that the recommendations provided here are based on our clinical experience in managing this patient population and on data from the literature addressing sarcopenia and osteoporosis in the context of CKD. These guidelines have not yet been codified in any official recommendations.

In fact, very often, the literature suggests methods that, although recognised as gold standards (e.g., DEXA and MRI for the evaluation of body composition), are however not used by most of common nutritional‐nephrological laboratories because they are not available, expensive, invasive and not repeatable within short periods.

Osteosarcopenia is a condition that results from the simultaneous presence of osteopenia and sarcopenia so the identification of this new syndrome lies in verifying the copresence of both conditions. It could be defined as

1. Osteopenia (*T*‐score ≤−1 and ≥−2.5 at the lumbar spine, femoral neck, total hip or distal 1/3 radius on DXA exam) combined with established sarcopenia (reduction in skeletal muscle mass and/or impairment in physical performance) [1, 6, 31]
2. Osteoporosis (*T*‐score ≤ −2.5 at the lumbar spine, femoral neck, total hip or distal 1/3 radius on DXA exam) and risk of sarcopenia (impairment in muscle strength, without established reduction in muscle mass and/or physical performance)
3. Copresence of established osteoporosis (*T*‐score ≤ −2.5 at the lumbar spine, femoral neck, total hip or distal 1/3 radius on DXA exam) and sarcopenia (reduction in skeletal muscle mass and/or impairment in physical performance)

These definitions, particularly the definition of osteoporosis, are also applicable to CKD patients. In fact, although CKD‐MBD presents a complex pathophysiology, the key factor for diagnosis ultimately remains bone density. When bone density is impaired, it indicates the presence of osteoporosis, regardless of whether this impairment is due to osteomalacia and/or vitamin D deficiency, both of which are characteristic features of CKD‐MBD [93].

**FIGURE 4:**
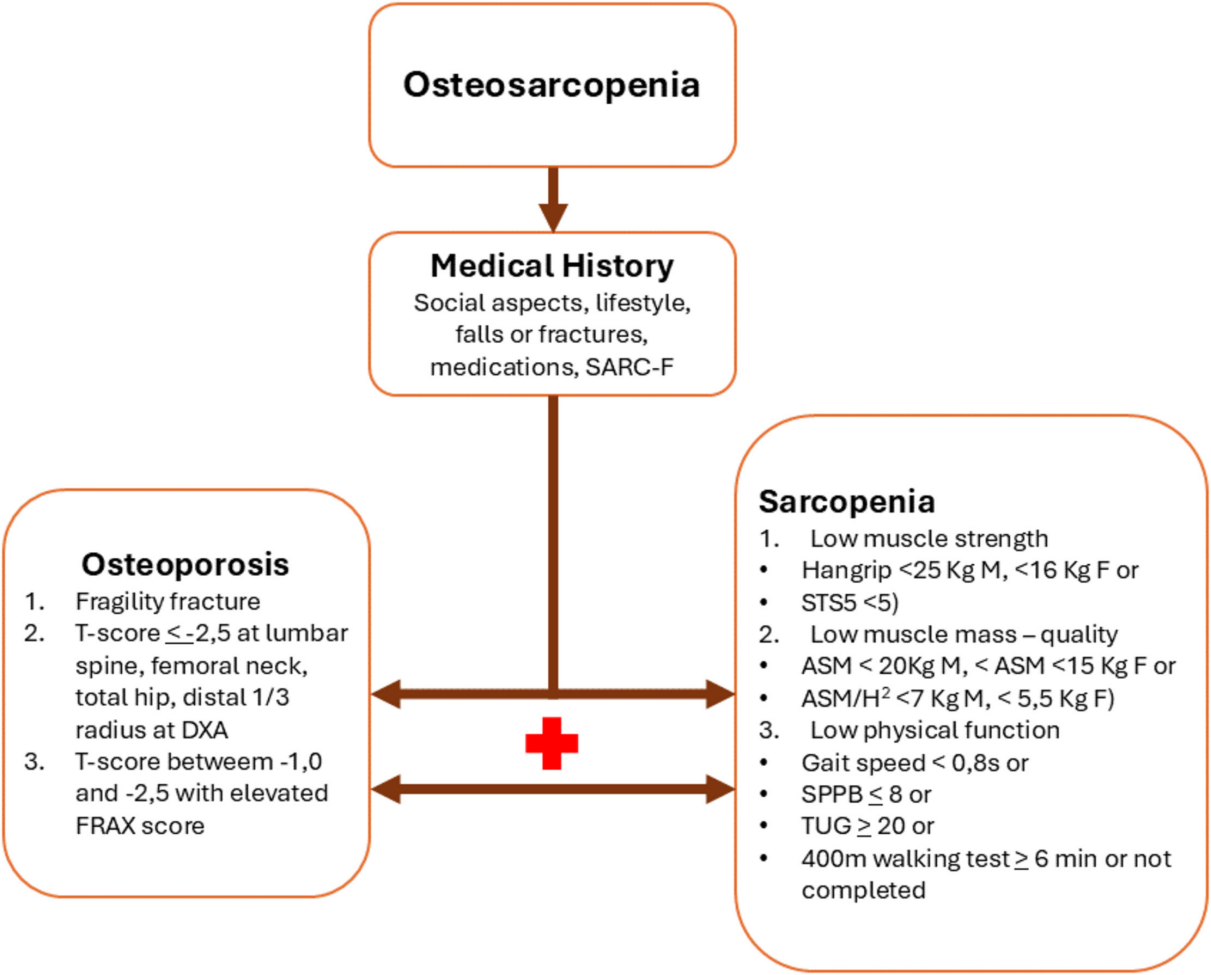
Flowchart to diagnose osteosarcopenia. ASM, appendicular skeletal muscle mass; DXA, dual X‐ray absorptiometry; F, female; FRAX, Fracture Risk Assessment Tool; M, male; SARC‐F, strength, assistance with walking, rising from a chair, climbing stairs and falls; SPPB, short physical performance battery; STS5, 5 times sit‐to‐stand; TUG, timed up‐and‐go.

Figure 4 suggests a possible path to follow for osteosarcopenia diagnosis. The first step for diagnosis is a careful history to identify a potential risk condition. In the case of elderly patients or those with cognitive problems, it is important to also involve family members and caregivers for a more complete collection of information. The history collects medical, social and lifestyle information and presence of previous falls and fractures. Since osteosarcopenia is a recently diagnosed syndrome, there are no data relating to the association of fractures and falls in CKD patients. According to the Network Europe Consensus for the prevention of falls [108], a fall is an event where a person comes to rest inadvertently on the ground or other lower level. To investigate falls occurrence, during history collection, patients should be asked ‘In the past 3 months, have you had any falls including a slip or trip in which you lost your balance and landed on the floor or ground or lowest level?’ [109].

During the medical history collection, it could be advisable to administer the SARC‐F questionnaire [110]. This tool encourages self‐reporting of signs suggestive and characteristic of sarcopenia, and it allows the detection of a potential risk of sarcopenia [98]. The SARC‐F is a 5‐item questionnaire that investigates as many components, strength, assistance in walking, rise from a chair, climb stairs and falls, that could be affected in the presence of sarcopenia. Each component has a score from 0 to 2 points, and the total score varies from 0 to 10 where 0 means ‘good’ and 10 means ‘worst’. SARC‐F was shown to be a valid tool to identify people at risk of sarcopenia [110]. Du et al. examined the validity of the SARC‐F questionnaire for screening sarcopenia among 105 CKD patients in conservative therapy and 125 patients on haemodialysis and found that it had low‐to‐moderate sensitivity but high specificity for screening sarcopenia among patients with CKD [111]. In any case, SARC‐F can be recommended as a simple method to apply in the first step of osteosarcopenia risk screening to identify patients who need subsequent specific investigations.

## 4 Translational Perspectives

The complex pathophysiology of osteosarcopenia in CKD is driven by hormonal dysregulation, chronic inflammation and metabolic dysfunction. Effective therapeutic strategies must target these multifactorial processes to prevent or minimise both bone and muscle loss.

A cornerstone of management is the treatment of CKD‐MBD [112]. This includes the use of phosphate binders and dietary phosphate restriction to control hyperphosphatemia, and vitamin D supplementation to improve calcium absorption and reduce PTH levels [112]. Calcimimetics further reduce bone turnover and the risk of osteoporosis by modulating calcium and phosphate homeostasis [112].

Given the important role of inflammation in osteosarcopenia, anti‐inflammatory interventions are crucial. Targeting pro‐inflammatory cytokines such as IL‐6 and TNF‐α with biological therapies or anti‐inflammatory agents, coupled with an anti‐inflammatory diet rich in omega‐3 fatty acids, can attenuate systemic inflammation, preserve lean muscle mass and support bone integrity [113].

Skeletal muscle maintenance is another important therapeutic focus. Resistance exercise is highly effective in increasing muscle mass, improving strength and stimulating bone formation [84]. Myostatin inhibitors, which promote muscle regeneration, offer an additional avenue for muscle preservation [114]. In addition, therapies targeting the growth hormone/insulin‐like growth factor 1 (GH/IGF‐1) axis may further stimulate muscle protein synthesis and bone formation, thus helping to counteract sarcopenia in CKD patients [115].

Sclerostin inhibition is a promising new approach to bone health. Sclerostin antibodies, such as romosozumab, improve bone formation by increasing osteoblast activity and addressing the low bone turnover often seen in CKD [116]. Hormone replacement therapies, including testosterone supplementation for hypogonadal men and oestrogen therapy for postmenopausal women, can also improve BMD and reduce fracture risk [116].

For patients with sarcopenic obesity, weight management and physical activity are essential components of treatment. Nutritional support, including tailored protein intake and supplementation with amino acids or creatine, plays an important role in promoting muscle anabolism and maintaining bone health, especially in malnourished CKD patients [117].

Finally, emerging therapies such as Klotho enhancement—via gene therapy or pharmacological agents—represent a promising frontier for protecting both muscle and bone health [118]. Overall, a comprehensive and individualised therapeutic approach integrating pharmacological, nutritional and physical interventions is essential to combat the adverse effects of osteosarcopenia and improve clinical outcomes in CKD patients.

## 5 Conclusion

In conclusion, osteosarcopenia has recently been recognised as a major complication in CKD, reflecting the convergence of osteoporosis (bone loss) and sarcopenia (muscle loss) in this population. This condition is gaining attention because of its multifactorial pathophysiology and significant impact on morbidity and mortality in CKD patients. The recognition of osteosarcopenia as a distinct clinical entity in CKD stems from increasing evidence highlighting the interrelationship between bone and muscle homeostasis, both of which are profoundly disrupted in CKD. Osteosarcopenia thus represents a new clinical symptom of CKD, driven by a combination of CKD‐related mineral disturbances, chronic inflammation, hormonal imbalances and malnutrition. Recognising and treating osteosarcopenia in CKD is essential to improve patient outcomes and reduce the risk of fractures, falls and disability.

This review highlights a need for comprehensive diagnostic strategies that address both bone and muscle health, especially using accessible and non‐invasive techniques such as MRI. The integration of these diagnostic tools in the nephrological‐nutritional setting is critical to the effective management and treatment of osteosarcopenia. Given the increasing prevalence of CKD and its associated complications, early detection and targeted intervention of osteosarcopenia could reduce the high morbidity and mortality associated with this condition. Ongoing research into the molecular mechanisms of osteosarcopenia and its treatment is essential to improve the quality of life of patients with CKD.

## Conflicts of Interest

Simone Vettoretti served as a consultant at advisory boards for Merk Sharp & Dohme, Astra Zeneca, Boehringer and Ingelheim and held a sponsored lecture by Dr. Shär. Lara Caldiroli worked as a consultant for Dr. Shär.

## References

[1] B. Kirk , J. Zanker , and G. Duque , “Osteosarcopenia: Epidemiology, Diagnosis, and Treatment‐Facts and Numbers,” Journal of Cachexia, Sarcopenia and Muscle 11 (2020): 609–618. 32202056 10.1002/jcsm.12567 PMC7296259

[2] R. M. Daly , B. E. Rosengren , G. Alwis , H. G. Ahlborg , I. Sernbo , and M. K. Karlsson , “Gender Specific Age‐Related Changes in Bone Density, Muscle Strength and Functional Performance in the Elderly: A‐10 Year Prospective Population‐Based Study,” BMC Geriatrics 13 (2013): 1–9. 23829776 10.1186/1471-2318-13-71 PMC3716823

[3] B. Kirk , A. Al Saedi , and G. Duque , “Osteosarcopenia: A Case of Geroscience,” Aging Medicine 2 (2019): 147–156. 31942528 10.1002/agm2.12080 PMC6880711

[4] A. J. Cruz‐Jentoft , G. Bahat , J. Bauer , et al., “Sarcopenia: Revised European Consensus on Definition and Diagnosis,” Age and Ageing 48 (2019): 16–31. 30312372 10.1093/ageing/afy169 PMC6322506

[5] P. Molinari , L. Caldiroli , E. Dozio , et al., “Association Between Advanced Glycation End‐Products and Sarcopenia in Patients With Chronic Kidney Disease,” Biomedicine 10 (2022): 1489. 10.3390/biomedicines10071489 PMC9313160 35884793

[6] H. P. Hirschfeld , R. Kinsella , and G. Duque , “Osteosarcopenia: Where Bone, Muscle, and Fat Collide,” Osteoporosis International 28 (2017): 2781–2790. 28733716 10.1007/s00198-017-4151-8

[7] E. A. Greco , P. Pietschmann , and S. Migliaccio , “Osteoporosis and Sarcopenia Increase Frailty Syndrome in the Elderly,” Frontiers in endocrinology 10 (2019): 255. 31068903 10.3389/fendo.2019.00255 PMC6491670

[8] B. R. Nielsen , J. Abdulla , H. E. Andersen , P. Schwarz , and C. Suetta , “Sarcopenia and Osteoporosis in Older People: A Systematic Review and Meta‐Analysis,” European Geriatric Medicine 9 (2018): 419–434. 34674498 10.1007/s41999-018-0079-6

[9] S. Sjöblom , J. Suuronen , T. Rikkonen , R. Honkanen , H. Kröger , and J. Sirola , “Relationship Between Postmenopausal Osteoporosis and the Components of Clinical Sarcopenia,” Maturitas 75 (2013): 175–180. 23628279 10.1016/j.maturitas.2013.03.016

[10] M. Locquet , C. Beaudart , J. Y. Reginster , and O. Bruyère , “Association Between the Decline in Muscle Health and the Decline in Bone Health in Older Individuals From the SarcoPhAge Cohort,” Calcified Tissue International 104 (2019): 273–284. 30511152 10.1007/s00223-018-0503-4

[11] R. M. Lima , R. J. de Oliveira , R. Raposo , S. G. R. Neri , and A. B. Gadelha , “Stages of Sarcopenia, Bone Mineral Density, and the Prevalence of Osteoporosis in Older Women,” Archives of Osteoporosis 14 (2019): 1–8. 10.1007/s11657-019-0591-4 30868338

[12] E. Bellorin‐Font , E. Rojas , and K. J. Martin , “Bone Disease in Chronic Kidney Disease and Kidney Transplant,” Nutrients 15 (2022): 167. 36615824 10.3390/nu15010167 PMC9824497

[13] P. Molinari , C. M. Alfieri , D. Mattinzoli , et al., “Bone and Mineral Disorder in Renal Transplant Patients: Overview of Pathology, Clinical, and Therapeutic Aspects,” Frontiers in Medicine 9 (2022): 821884. 35360722 10.3389/fmed.2022.821884 PMC8960161

[14] J. B. Cannata‐Andía , B. Martín‐Carro , J. Martín‐Vírgala , et al., “Chronic Kidney Disease‐Mineral and Bone Disorders: Pathogenesis and Management,” Calcified Tissue International 108 (2021): 410–422. 33190187 10.1007/s00223-020-00777-1

[15] K. A. Hruska , T. Sugatani , O. Agapova , and Y. Fang , “The Chronic Kidney Disease—Mineral Bone Disorder (CKD‐MBD): Advances in Pathophysiology,” Bone 100 (2017): 80–86. 28119179 10.1016/j.bone.2017.01.023 PMC5502716

[16] T. Isakova , P. Wahl , G. S. Vargas , et al., “Fibroblast Growth Factor 23 is Elevated Before Parathyroid Hormone and Phosphate in Chronic Kidney Disease,” Kidney International 79 (2011): 1370–1378. 21389978 10.1038/ki.2011.47 PMC3134393

[17] J. Botelho , V. Machado , L. Proença , A. S. Delgado , and J. J. Mendes , “Vitamin D Deficiency and Oral Health: A Comprehensive Review,” Nutrients 12 (2020): 1471. 32438644 10.3390/nu12051471 PMC7285165

[18] P. P. Centeno , A. Herberger , H. C. Mun , et al., “Phosphate Acts Directly on the Calcium‐Sensing Receptor to Stimulate Parathyroid Hormone Secretion,” Nature Communications 10 (2019): 4693. 10.1038/s41467-019-12399-9 PMC6795806 31619668

[19] E. Gielen , J. Dupont , M. Dejaeger , and M. R. Laurent , “Sarcopenia, Osteoporosis and Frailty,” Metabolism 145 (2023): 155638. 37348597 10.1016/j.metabol.2023.155638

[20] Q. Wang , J. Zhao , H. Chen , et al., “Bmi‐1 Overexpression Improves Sarcopenia Induced by 1,25(OH)2 D3 Deficiency and Downregulates GATA4‐Dependent Rela Transcription,” Journal of Bone and Mineral Research 38 (2023): 427–442. 36625422 10.1002/jbmr.4770

[21] C. M. Girgis , K. M. Cha , B. So , et al., “Mice With Myocyte Deletion of Vitamin D Receptor Have Sarcopenia and Impaired Muscle Function,” Journal of Cachexia, Sarcopenia and Muscle 10 (2019): 1228–1240. 31225722 10.1002/jcsm.12460 PMC6903451

[22] S. Sakai , M. Suzuki , Y. Tashiro , et al., “Vitamin D Receptor Signaling Enhances Locomotive Ability in Mice,” Journal of Bone and Mineral Research 30 (2015): 128–136. 25043694 10.1002/jbmr.2317

[23] R. M. Elias , M. A. Dalboni , A. C. E. Coelho , and R. M. A. Moysés , “CKD‐MBD: From the Pathogenesis to the Identification and Development of Potential Novel Therapeutic Targets,” Current Osteoporosis Reports 16 (2018): 693–702. 30291515 10.1007/s11914-018-0486-0

[24] D. Mattinzoli , P. Molinari , G. Romero‐González , et al., “Is There a Role in Acute Kidney Injury for FGF23 and Klotho?,” Clinical Kidney Journal 16 (2023): 1555–1562. 37779849 10.1093/ckj/sfad093 PMC10539225

[25] D. Mattinzoli , M. Ikehata , K. Tsugawa , et al., “FGF23 and Fetuin‐A Interaction and Mesenchymal Osteogenic Transformation,” International Journal of Molecular Sciences 20 (2019): 915. 30791553 10.3390/ijms20040915 PMC6412477

[26] D. Mattinzoli , M. P. Rastaldi , M. Ikehata , et al., “FGF23‐Regulated Production of Fetuin‐A (AHSG) in Osteocytes,” Bone 83 (2016): 35–47. 26476373 10.1016/j.bone.2015.10.008

[27] F. G. Graciolli , K. R. Neves , F. Barreto , et al., “The Complexity of Chronic Kidney Disease‐Mineral and Bone Disorder Across Stages of Chronic Kidney Disease,” Kidney International 91 (2017): 1436–1446. 28318623 10.1016/j.kint.2016.12.029

[28] M. F. P. Santos , M. J. Hernández , I. B. de Oliveira , et al., “Comparison of Clinical, Biochemical and Histomorphometric Analysis of Bone Biopsies in Dialysis Patients With and Without Fractures,” Journal of Bone and Mineral Metabolism 37 (2019): 125–133. 29372334 10.1007/s00774-018-0902-7

[29] C. Alfieri , V. Binda , S. Malvica , et al., “Bone Effect and Safety of One‐Year Denosumab Therapy in a Cohort of Renal Transplanted Patients: An Observational Monocentric Study,” Journal of Clinical Medicine 10 (2021): 1989. 34066345 10.3390/jcm10091989 PMC8124304

[30] A. L. H. Huynh , S. T. Baker , A. J. Stewardson , and D. F. Johnson , “Denosumab‐Associated Hypocalcaemia: Incidence, Severity and Patient Characteristics in a Tertiary Hospital Setting,” Pharmacoepidemiology and Drug Safety 25 (2016): 1274–1278. 27255807 10.1002/pds.4045

[31] Y. Wang , W. Ma , J. Pu , and F. Chen , “Interrelationships Between Sarcopenia, Bone Turnover Markers and Low Bone Mineral Density in Patients on Hemodialysis,” Renal Failure 45 (2023): 2200846. 37122165 10.1080/0886022X.2023.2200846 PMC10134952

[32] KDIGO 2017 Clinical Practice Guideline Update for the Diagnosis, Evaluation, Prevention, and Treatment of Chronic Kidney Disease‐Mineral and Bone Disorder (CKD‐MBD),” Kidney International. Supplement 7 (2017): 1–59. 10.1016/j.kisu.2017.04.001 PMC6340919 30675420

[33] A. Polito , L. Barnaba , D. Ciarapica , and E. Azzini , “Osteosarcopenia: A Narrative Review on Clinical Studies,” International Journal of Molecular Sciences 23 (2022): 5591. 35628399 10.3390/ijms23105591 PMC9147376

[34] B. R. Nielsen , J. Abdulla , H. E. Andersen , P. Schwarz , and C. Suetta , “Sarcopenia and Osteoporosis in Older People: A Systematic Review and Meta‐Analysis,” Eur Geriatr Med 9 (2018): 419–434. 34674498 10.1007/s41999-018-0079-6

[35] M. Maravic , A. Ostertag , P. U. Torres , and M. Cohen‐Solal , “Incidence and Risk Factors for Hip Fractures in Dialysis Patients,” Osteoporosis International 25 (2014): 159–165. 23835863 10.1007/s00198-013-2435-1

[36] F. Tentori , K. McCullough , R. D. Kilpatrick , et al., “Response to High Rates of Death and Hospitalization Follow Bone Fracture Among Hemodialysis Patients,” Kidney International 85 (2013): 166–173. 10.1038/ki.2013.279. PMC4141532 25152548

[37] J. Paintin , C. Cooper , and E. Dennison , “Osteosarcopenia,” British Journal of Hospital Medicine 79 (2018): 253–258. 29727228 10.12968/hmed.2018.79.5.253 PMC5963675

[38] A. Slee , C. McKeaveney , G. Adamson , et al., “Estimating the Prevalence of Muscle Wasting, Weakness, and Sarcopenia in Hemodialysis Patients,” Journal of Renal Nutrition 30 (2020): 313–321. 31734056 10.1053/j.jrn.2019.09.004

[39] A. Davenport , “Frailty, Appendicular Lean Mass, Osteoporosis and Osteosarcopenia in Peritoneal Dialysis Patients,” Journal of Nephrology 35 (2022): 2333–2340. 35816240 10.1007/s40620-022-01390-1 PMC9700626

[40] S. Sridharan , E. Vilar , S. Ramanarayanan , A. Davenport , and K. Farrington , “Energy Expenditure Estimates in Chronic Kidney Disease Using a Novel Physical Activity Questionnaire,” Nephrology, Dialysis, Transplantation 37 (2022): 515–521. 10.1093/ndt/gfaa377 33416874

[41] A. Davenport , “Application of the Clinical Frailty Score and Body Composition and Upper Arm Strength in Haemodialysis Patients,” Clinical Kidney Journal 15 (2021): 553–559. 35211309 10.1093/ckj/sfab228 PMC8862041

[42] M. I. B. Silva , K. Picard , and M. R. S. T. Klein , “Sarcopenia and Sarcopenic Obesity in Chronic Kidney Disease: Update on Prevalence, Outcomes, Risk Factors and Nutrition Treatment,” Current Opinion in Clinical Nutrition and Metabolic Care 25 (2022): 371–377. 36039925 10.1097/MCO.0000000000000871

[43] T. Ji , Y. Li , and L. Ma , “Sarcopenic Obesity: An Emerging Public Health Problem,” Aging and Disease 13 (2022): 379–388. 35371597 10.14336/AD.2021.1006 PMC8947824

[44] R. N. Baumgartner , “Body Composition in Healthy Aging,” Annals of the New York Academy of Sciences 904 (2000): 437–448. 10865787 10.1111/j.1749-6632.2000.tb06498.x

[45] F. Petermann‐Rocha , S. Yang , S. R. Gray , J. P. Pell , C. Celis‐Morales , and F. K. Ho , “Sarcopenic Obesity and Its Association With Respiratory Disease Incidence and Mortality—Authors’ Reply,” Clinical Nutrition 40 (2021): 2520. 33932797 10.1016/j.clnu.2021.03.030

[46] J. L. Atkins and S. G. Wannamathee , “Sarcopenic Obesity in Ageing: Cardiovascular Outcomes and Mortality,” British Journal of Nutrition 124 (2020): 1102–1113. 32616084 10.1017/S0007114520002172

[47] C. Koliaki , S. Liatis , M. Dalamaga , and A. Kokkinos , “Sarcopenic Obesity: Epidemiologic Evidence, Pathophysiology, and Therapeutic Perspectives,” Current Obesity Reports 8 (2019): 458–471. 31654335 10.1007/s13679-019-00359-9

[48] D. Scott , M. Seibel , R. Cumming , et al., “Sarcopenic Obesity and Its Temporal Associations With Changes in Bone Mineral Density, Incident Falls, and Fractures in Older Men: The Concord Health and Ageing in Men Project,” Journal of Bone and Mineral Research 32 (2017): 575–583. 27736026 10.1002/jbmr.3016

[49] M. R. Laurent , M. J. Cook , E. Gielen , et al., “Lower Bone Turnover and Relative Bone Deficits in Men With Metabolic Syndrome: A Matter of Insulin Sensitivity? The European Male Ageing Study,” Osteoporosis International 27 (2016): 3227–3237. 27273111 10.1007/s00198-016-3656-x

[50] J. Z. Ilich , O. J. Kelly , J. E. Inglis , L. B. Panton , G. Duque , and M. J. Ormsbee , “Interrelationship Among Muscle, Fat, and Bone: Connecting the Dots on Cellular, Hormonal, and Whole Body Levels,” Ageing Research Reviews 15 (2014): 51–60. 24632496 10.1016/j.arr.2014.02.007

[51] Q. Chen , P. Shou , C. Zheng , et al., “Fate Decision of Mesenchymal Stem Cells: Adipocytes or Osteoblasts?,” Cell Death and Differentiation 23 (2016): 1128–1139. 26868907 10.1038/cdd.2015.168 PMC4946886

[52] L. Singh , S. Tyagi , D. Myers , and G. Duque , “Good, Bad, or Ugly: The Biological Roles of Bone Marrow Fat,” Current Osteoporosis Reports 16 (2018): 130–137. 29476394 10.1007/s11914-018-0427-y

[53] C. S. Carter , J. N. Justice , and L. D. Thompson , “Lipotoxicity, Aging, and Muscle Contractility: Does Fiber Type Matter?,” Geroscience 41 (2019): 297–308. 31227962 10.1007/s11357-019-00077-z PMC6702511

[54] D. A. Rivas , D. J. McDonald , N. P. Rice , P. H. Haran , G. G. Dolnikowski , and R. A. Fielding , “Diminished Anabolic Signaling Response to Insulin Induced by Intramuscular Lipid Accumulation Is Associated With Inflammation in Aging but Not Obesity,” American Journal of Physiology. Regulatory, Integrative and Comparative Physiology 310 (2016): R561–R569. 26764052 10.1152/ajpregu.00198.2015 PMC4867383

[55] M. W. Hamrick , M. E. McGee‐Lawrence , and D. M. Frechette , “Fatty Infiltration of Skeletal Muscle: Mechanisms and Comparisons With Bone Marrow Adiposity,” Frontiers in Endocrinology 7 (2016): 69. 27379021 10.3389/fendo.2016.00069 PMC4913107

[56] E. Roh and K. M. Choi , “Health Consequences of Sarcopenic Obesity: A Narrative Review,” Frontiers in endocrinology 11 (2020): 332. 32508753 10.3389/fendo.2020.00332 PMC7253580

[57] M. I. Barreto Silva , K. Picard , and M. R. S. T. Klein , “Sarcopenia and Sarcopenic Obesity in Chronic Kidney Disease: Update on Prevalence, Outcomes, Risk Factors and Nutrition Treatment,” Current Opinion in Clinical Nutrition and Metabolic Care 25 (2022): 371–377. 36039925 10.1097/MCO.0000000000000871

[58] L. Ozbek , S. M. Abdel‐Rahman , S. Unlu , et al., “Exploring Adiposity and Chronic Kidney Disease: Clinical Implications, Management Strategies, Prognostic Considerations,” Medicina (Kaunas, Lithuania) 60 (2024): 1668. 39459455 10.3390/medicina60101668 PMC11509396

[59] L. Caldiroli , S. Vettoretti , S. Armelloni , et al., “Possible Benefits of a Low Protein Diet in Older Patients With CKD at Risk of Malnutrition: A Pilot Randomized Controlled Trial,” Frontiers in Nutrition 8 (2022): 8. 10.3389/fnut.2021.782499 PMC8860492 35198584

[60] P. Stenvinkel , O. Heimbürger , B. Lindholm , G. A. Kaysen , and J. Bergström , “Are There Two Types of Malnutrition in Chronic Renal Failure? Evidence for Relationships Between Malnutrition, Inflammation and Atherosclerosis (MIA Syndrome),” Nephrology, Dialysis, Transplantation 15 (2000): 953–960. 10.1093/ndt/15.7.953 10862630

[61] C. Franceschi , M. Bonafè , S. Valensin , et al., “Inflamm‐Aging: An Evolutionary Perspective on Immunosenescence,” Annals of the New York Academy of Sciences 908 (2000): 244–254. 10911963 10.1111/j.1749-6632.2000.tb06651.x

[62] M. A. Clynes , C. L. Gregson , O. Bruyère , C. Cooper , and E. M. Dennison , “Osteosarcopenia: Where Osteoporosis and Sarcopenia Collide,” Rheumatology (Oxford) 60 (2021): 529–537. 33276373 10.1093/rheumatology/keaa755

[63] E. S. Cannizzo , C. C. Clement , R. Sahu , C. Follo , and L. Santambrogio , “Oxidative Stress, Inflamm‐Aging and Immunosenescence,” Journal of Proteomics 74 (2011): 2313–2323. 21718814 10.1016/j.jprot.2011.06.005

[64] L. Holm , J. L. Olesen , K. Matsumoto , et al., “Protein‐Containing Nutrient Supplementation Following Strength Training Enhances the Effect on Muscle Mass, Strength, and Bone Formation in Postmenopausal Women,” Journal of Applied Physiology 105 (2008): 274–281. 18467544 10.1152/japplphysiol.00935.2007

[65] E. Curtis , A. Litwic , C. Cooper , and E. Dennison , “Determinants of Muscle and Bone Aging,” Journal of Cellular Physiology 230 (2015): 2618–2625. 25820482 10.1002/jcp.25001 PMC4530476

[66] R. R. McLean , “Proinflammatory Cytokines and Osteoporosis,” Current Osteoporosis Reports 7 (2009): 134–139. 19968917 10.1007/s11914-009-0023-2

[67] K. Dahl , L. A. Ahmed , R. M. Joakimsen , et al., “High‐Sensitivity C‐Reactive Protein Is an Independent Risk Factor for Non‐Vertebral Fractures in Women and Men: The Tromsø Study,” Bone 72 (2015): 65–70. 25460573 10.1016/j.bone.2014.11.012

[68] S. Ishii , J. A. Cauley , G. A. Greendale , et al., “C‐Reactive Protein, Bone Strength, and Nine‐Year Fracture Risk: Data From the Study of Women's Health Across the Nation (SWAN),” Journal of Bone and Mineral Research 28 (2013): 1688–1698. 23456822 10.1002/jbmr.1915 PMC3880424

[69] H. H. Ting and K. H. Long , “Hospital Performance and Acute Coronary Syndrome Outcomes,” JAMA 296 (2006): 1349. 16985222 10.1001/jama.296.11.1349-a

[70] J. A. Cauley , K. E. Barbour , S. L. Harrison , et al., “Inflammatory Markers and the Risk of Hip and Vertebral Fractures in Men: The Osteoporotic Fractures in Men (MrOS),” Journal of Bone and Mineral Research 31 (2016): 2129–2138. 27371811 10.1002/jbmr.2905 PMC5240475

[71] M. Bowser , S. Herberg , P. Arounleut , et al., “Effects of the Activin A‐Myostatin‐Follistatin System on Aging Bone and Muscle Progenitor Cells,” Experimental Gerontology 48 (2013): 290–297. 23178301 10.1016/j.exger.2012.11.004 PMC3678732

[72] A. Oliveira and C. Vaz , “The Role of Sarcopenia in the Risk of Osteoporotic Hip Fracture,” Clinical Rheumatology 34 (2015): 1673–1680. 25912213 10.1007/s10067-015-2943-9

[73] N. Gharahdaghi , B. E. Phillips , N. J. Szewczyk , K. Smith , D. J. Wilkinson , and P. J. Atherton , “Links Between Testosterone, Oestrogen, and the Growth Hormone/Insulin‐Like Growth Factor Axis and Resistance Exercise Muscle Adaptations,” Frontiers in Physiology 11 (2021): 621226. 33519525 10.3389/fphys.2020.621226 PMC7844366

[74] R. Skiba , A. Matyjek , T. Syryło , S. Niemczyk , and A. Rymarz , “Advanced Chronic Kidney Disease Is a Strong Predictor of Hypogonadism and Is Associated With Decreased Lean Tissue Mass,” International Journal of Nephrology and Renovascular Disease 13 (2020): 319–327. 33192085 10.2147/IJNRD.S275554 PMC7653405

[75] T. Morioka , “Myostatin: The Missing Link Between Sarcopenia and Cardiovascular Disease in Chronic Kidney Disease?,” Journal of Atherosclerosis and Thrombosis 27 (2020): 1036–1038. 32435012 10.5551/jat.ED129 PMC7585907

[76] M. R. Laurent , F. Jardí , V. Dubois , et al., “Androgens Have Antiresorptive Effects on Trabecular Disuse Osteopenia Independent From Muscle Atrophy,” Bone 93 (2016): 33–42. 27622887 10.1016/j.bone.2016.09.011

[77] T. Hosoi , M. Yakabe , H. Sasakawa , et al., “Sarcopenia Phenotype and Impaired Muscle Function in Male Mice With Fast‐Twitch Muscle‐Specific Knockout of the Androgen Receptor,” Proceedings of the National Academy of Sciences of the United States of America 120 (2023): e2218032120. 36669097 10.1073/pnas.2218032120 PMC9942915

[78] M. R. Laurent , L. Dedeyne , J. Dupont , B. Mellaerts , M. Dejaeger , and E. Gielen , “Age‐Related Bone Loss and Sarcopenia in Men,” Maturitas 122 (2019): 51–56. 30797530 10.1016/j.maturitas.2019.01.006

[79] M. Sinnesael , F. Jardi , L. Deboel , et al., “The Androgen Receptor Has No Direct Antiresorptive Actions in Mouse Osteoclasts,” Molecular and Cellular Endocrinology 411 (2015): 198–206. 25958043 10.1016/j.mce.2015.04.030

[80] F. Jardí , N. Kim , M. R. Laurent , et al., “Androgen Receptor in Neurons Slows Age‐Related Cortical Thinning in Male Mice,” Journal of Bone and Mineral Research 34 (2019): 508–519. 30496619 10.1002/jbmr.3625

[81] A. Mandelli , E. Tacconi , I. Levinger , G. Duque , and A. Hayes , “The Role of Estrogens in Osteosarcopenia: From Biology to Potential Dual Therapeutic Effects,” Climacteric 25 (2022): 81–87. 34423690 10.1080/13697137.2021.1965118

[82] S. Ali and N. N. Dave , “Sexual Dysfunction in Women With Kidney Disease,” Advances in Chronic Kidney Disease 27 (2020): 506–515. 33328067 10.1053/j.ackd.2020.07.005

[83] M. Farahmand , F. Ramezani Tehrani , D. Khalili , L. Cheraghi , and F. Azizi , “Endogenous Estrogen Exposure and Chronic Kidney Disease; A 15‐Year Prospective Cohort Study,” BMC Endocrine Disorders 21 (2021): 155. 34348694 10.1186/s12902-021-00817-3 PMC8336110

[84] F. Mallamaci , A. Pisano , and G. Tripepi , “Physical Activity in Chronic Kidney Disease and the EXerCise Introduction To Enhance Trial,” Nephrology, Dialysis, Transplantation 35 (2020): ii18–ii22. 10.1093/ndt/gfaa012 PMC7066543 32162664

[85] K. L. Johansen , “Exercise in the End‐Stage Renal Disease Population,” Journal of the American Society of Nephrology 18 (2007): 1845–1854. 17442789 10.1681/ASN.2007010009

[86] D. F. Cardoso , E. A. Marques , D. V. Leal , et al., “Impact of Physical Activity and Exercise on Bone Health in Patients With Chronic Kidney Disease: A Systematic Review of Observational and Experimental Studies,” BMC Nephrology 21 (2020): 334. 32770949 10.1186/s12882-020-01999-z PMC7414574

[87] X. H. Wang and W. E. Mitch , “Muscle Wasting From Kidney Failure—A Model for Catabolic Conditions,” International Journal of Biochemistry & Cell Biology 45 (2013): 2230–2238. 23872437 10.1016/j.biocel.2013.06.027 PMC3919551

[88] O. M. Akchurin and F. Kaskel , “Update on Inflammation in Chronic Kidney Disease,” Blood Purification 39 (2015): 84–92. 25662331 10.1159/000368940

[89] S. A. Sabri , J. C. Chavarria , C. Ackert‐Bicknell , C. Swanson , and E. Burger , “Osteoporosis: An Update on Screening, Diagnosis, Evaluation, and Treatment,” Orthopedics 46 (2023): E20–E26. 35876780 10.3928/01477447-20220719-03 PMC10084730

[90] N. B. Watts , P. M. Camacho , E. M. Lewiecki , and S. M. Petak , “American Association of Clinical Endocrinologists/American College of Endocrinology Clinical Practice Guidelines for the Diagnosis and Treatment of Postmenopausal Osteoporosis‐2020 Update,” Endocrine Practice 27 (2021): 379–380. 33577971 10.1016/j.eprac.2021.02.001

[91] R. H. Evans , S. J. Evans , P. C. Pook , and D. C. Sunter , “A Comparison of Excitatory Amino Acid Antagonists Acting at Primary Afferent C Fibres and Motoneurones of the Isolated Spinal Cord of the Rat,” British Journal of Pharmacology 91 (1987): 531–537. 3038242 10.1111/j.1476-5381.1987.tb11246.x PMC1853540

[92] F. Cosman , S. J. de Beur , M. S. LeBoff , et al., “Clinician's Guide to Prevention and Treatment of Osteoporosis,” Osteoporosis International 25 (2014): 2359–2381. 25182228 10.1007/s00198-014-2794-2 PMC4176573

[93] P. M. Camacho , S. M. Petak , N. Binkley , et al., “American Association of Clinical Endocrinologists and American College of Endocrinology Clinical Practice Guidelines for the Diagnosis and Treatment of Postmenopausal Osteoporosis—2016—Executive Summary,” Endocrine Practice 22 (2016): 1111–1118. 27643923 10.4158/EP161435.ESGL

[94] M. Ketteler , G. A. Block , P. Evenepoel , et al., “Diagnosis, Evaluation, Prevention, and Treatment of Chronic Kidney Disease‐Mineral and Bone Disorder: Synopsis of the Kidney Disease: Improving Global Outcomes 2017 Clinical Practice Guideline Update,” Annals of Internal Medicine 168 (2018): 422–430. 29459980 10.7326/M17-2640

[95] K. Sridharan , “Chronic Kidney Disease Mineral and Bone Disorder: A Guide for General Practice,” Australian Journal of General Practice 52 (2023): 52–57. 36796773 10.31128/AJGP-03-22-6365

[96] H. Ogata , H. Sugawara , M. Yamamoto , and H. Ito , “Phosphate and Coronary Artery Disease in Patients With Chronic Kidney Disease,” Journal of Atherosclerosis and Thrombosis 31 (2024): 1–14. 37766573 10.5551/jat.RV22012 PMC10776333

[97] L. Hu , A. Napoletano , M. Provenzano , et al., “Mineral Bone Disorders in Kidney Disease Patients: The Ever‐Current Topic,” International Journal of Molecular Sciences 23 (2022): 12223. 36293076 10.3390/ijms232012223 PMC9603742

[98] A. J. Cruz‐Jentoft , G. Bahat , J. Bauer , et al., “Sarcopenia: Revised European Consensus on Definition and Diagnosis,” Age and Ageing 48 (2019): 16–31. 30312372 10.1093/ageing/afy169 PMC6322506

[99] W. Nijholt , A. Scafoglieri , H. Jager‐Wittenaar , J. S. M. Hobbelen , and C. P. van der Schans , “The Reliability and Validity of Ultrasound to Quantify Muscles in Older Adults: A Systematic Review,” Journal of Cachexia, Sarcopenia and Muscle 8 (2017): 702–712. 28703496 10.1002/jcsm.12210 PMC5659048

[100] K. J. Lambell , A. C. Tierney , J. C. Wang , et al., “Comparison of Ultrasound‐Derived Muscle Thickness With Computed Tomography Muscle Cross‐Sectional Area on Admission to the Intensive Care Unit: A Pilot Cross‐Sectional Study,” JPEN Journal of Parenteral and Enteral Nutrition 45 (2021): 136–145. 32291773 10.1002/jpen.1822

[101] A. Sabatino , U. Maggiore , G. Regolisti , et al., “Ultrasound for Non‐Invasive Assessment and Monitoring of Quadriceps Muscle Thickness in Critically Ill Patients With Acute Kidney Injury,” Frontiers in Nutrition 8 (2021): 622823. 33937303 10.3389/fnut.2021.622823 PMC8081900

[102] J. Lortie , B. Rush , K. Osterbauer , et al., “Myosteatosis as a Shared Biomarker for Sarcopenia and Cachexia Using MRI and Ultrasound,” Frontiers in Rehabilitation Sciences 3 (2022): 896114. 36189019 10.3389/fresc.2022.896114 PMC9397668

[103] T. J. Wilkinson , L. A. Baker , E. L. Watson , et al., “Skeletal Muscle Texture Assessment Using Ultrasonography: Comparison With Magnetic Resonance Imaging in Chronic Kidney Disease,” Ultrasonic Imaging 46 (2024): 263–268. 38807343 10.1177/01617346241255879 PMC11325600

[104] J. J. Carrero , P. Stenvinkel , L. Cuppari , et al., “Etiology of the Protein‐Energy Wasting Syndrome in Chronic Kidney Disease: A Consensus Statement From the International Society of Renal Nutrition and Metabolism (ISRNM),” Journal of Renal Nutrition 23 (2013): 77–90. 23428357 10.1053/j.jrn.2013.01.001

[105] T. A. Ikizler , J. D. Burrowes , L. D. Byham‐Gray , et al., “KDOQI Clinical Practice Guideline for Nutrition in CKD: 2020 Update,” American Journal of Kidney Diseases 76 (2020): S1–S107. 32829751 10.1053/j.ajkd.2020.05.006

[106] A. Sabatino , L. Cuppari , P. Stenvinkel , B. Lindholm , and C. M. Avesani , “Sarcopenia in Chronic Kidney Disease: What Have We Learned so Far?,” Journal of Nephrology 34 (2021): 1347–1372. 32876940 10.1007/s40620-020-00840-y PMC8357704

[107] V. A. de Souza , D. Oliveira , S. R. Barbosa , et al., “Sarcopenia in Patients With Chronic Kidney Disease Not yet on Dialysis: Analysis of the Prevalence and Associated Factors,” PLoS ONE 12 (2017): e0176230. 28448584 10.1371/journal.pone.0176230 PMC5407780

[108] S. E. Lamb , E. C. Jørstad‐Stein , K. Hauer , and C. Becker , “Development of a Common Outcome Data Set for Fall Injury Prevention Trials: The Prevention of Falls Network Europe Consensus,” Journal of the American Geriatrics Society 53 (2005): 1618–1622. 16137297 10.1111/j.1532-5415.2005.53455.x

[109] M. P. Duarte , M. S. Pereira , V. M. Baião , et al., “Design and Methodology of the SARCopenia Trajectories and Associations With Adverse Clinical Outcomes in Patients on HemoDialysis: The SARC‐HD Study,” BMC Nephrology 24 (2023): 239. 37582699 10.1186/s12882-023-03168-4 PMC10428584

[110] T. K. Malmstrom , D. K. Miller , E. M. Simonsick , L. Ferrucci , and J. E. Morley , “SARC‐F: A Symptom Score to Predict Persons With Sarcopenia at Risk for Poor Functional Outcomes,” Journal of Cachexia, Sarcopenia and Muscle 7 (2016): 28–36. 27066316 10.1002/jcsm.12048 PMC4799853

[111] W. Du , C. Gao , X. Wang , et al., “Validity of the SARC‐F Questionnaire in Assessing Sarcopenia in Patients With Chronic Kidney Disease: A Cross‐Sectional Study,” Frontiers in Medicine 10 (2023): 1188971. 37534318 10.3389/fmed.2023.1188971 PMC10391647

[112] M. Fatima , S. L. Brennan‐Olsen , and G. Duque , “Therapeutic Approaches to Osteosarcopenia: Insights for the Clinician,” Therapeutic Advances in Musculoskeletal Disease 11 (2019): 1759720X19867009. 10.1177/1759720X19867009 PMC6686316 31431811

[113] C. McGlory , P. C. Calder , and E. A. Nunes , “The Influence of Omega‐3 Fatty Acids on Skeletal Muscle Protein Turnover in Health, Disuse, and Disease,” Frontiers in Nutrition 6 (2019): 144. 31555658 10.3389/fnut.2019.00144 PMC6742725

[114] D. Verzola , C. Barisione , D. Picciotto , G. Garibotto , and L. Koppe , “Emerging Role of Myostatin and Its Inhibition in the Setting of Chronic Kidney Disease,” Kidney International 95 (2019): 506–517. 30598193 10.1016/j.kint.2018.10.010

[115] P. Kamenický , G. Mazziotti , M. Lombès , A. Giustina , and P. Chanson , “Growth Hormone, Insulin‐Like Growth Factor‐1, and the Kidney: Pathophysiological and Clinical Implications,” Endocrine Reviews 35 (2014): 234–281. 24423979 10.1210/er.2013-1071

[116] A. Moretti and G. Iolascon , “Sclerostin: Clinical Insights in Muscle‐Bone Crosstalk,” Journal of International Medical Research 51 (2023): 3000605231193293. 37632438 10.1177/03000605231193293 PMC10467411

[117] C. A. M. Anderson and H. A. Nguyen , “Nutrition Education in the Care of Patients With Chronic Kidney Disease and End‐Stage Renal Disease,” Seminars in Dialysis 31 (2018): 115–121. 29455475 10.1111/sdi.12681

[118] M. R. Haussler , G. K. Whitfield , C. A. Haussler , et al., “1,25‐Dihydroxyvitamin D and Klotho: A Tale of Two Renal Hormones Coming of Age,” Vitamins and Hormones 100 (2016): 165–230. 26827953 10.1016/bs.vh.2015.11.005

